# Applying Independent Component Analysis to Clinical fMRI at 7 T

**DOI:** 10.3389/fnhum.2013.00496

**Published:** 2013-09-02

**Authors:** Simon Daniel Robinson, Veronika Schöpf, Pedro Cardoso, Alexander Geissler, Florian Ph. S. Fischmeister, Moritz Wurnig, Siegfried Trattnig, Roland Beisteiner

**Affiliations:** ^1^High Field Magnetic Resonance Imaging Centre of Excellence, Medical University of Vienna, Vienna, Austria; ^2^Department of Radiology, Medical University of Vienna, Vienna, Austria; ^3^Study Group Clinical fMRI, Department of Neurology, Medical University of Vienna, Vienna, Austria

**Keywords:** independent component analysis, ultra-high field fMRI, presurgical planning, motor, neurology, motion, artifacts

## Abstract

Increased BOLD sensitivity at 7 T offers the possibility to increase the reliability of fMRI, but ultra-high field is also associated with an increase in artifacts related to head motion, Nyquist ghosting, and parallel imaging reconstruction errors. In this study, the ability of independent component analysis (ICA) to separate activation from these artifacts was assessed in a 7 T study of neurological patients performing chin and hand motor tasks. ICA was able to isolate primary motor activation with negligible contamination by motion effects. The results of General Linear Model (GLM) analysis of these data were, in contrast, heavily contaminated by motion. Secondary motor areas, basal ganglia, and thalamus involvement were apparent in ICA results, but there was low capability to isolate activation in the same brain regions in the GLM analysis, indicating that ICA was more sensitive as well as more specific. A method was developed to simplify the assessment of the large number of independent components. Task-related activation components could be automatically identified via these intuitive and effective features. These findings demonstrate that ICA is a practical and sensitive analysis approach in high field fMRI studies, particularly where motion is evoked. Promising applications of ICA in clinical fMRI include presurgical planning and the study of pathologies affecting subcortical brain areas.

## Introduction

Time-series SNR and BOLD sensitivity (BS) increase with field strength (Triantafyllou et al., 2005; van der Zwaag et al., 2009; Beisteiner et al., 2011; Duchin et al., 2012), motivating the use of very high field for fMRI (Barth and Poser, 2011; De Martino et al., 2011; Ugurbil, 2012). In clinical fMRI applications such as presurgical planning (Roessler et al., 2005; Stippich, 2007) with a patient cohort that may have limited tolerance in an fMRI session, increased BS may allow the measurement time to be reduced, or the reliability of fMRI findings to be increased for a particular measurement time. Against this prospect of increased sensitivity at ultra-high field stand a number of methodical challenges. In clinical practice, the most significant of these is increased head motion artifacts. Motion artifacts are the most frequent reason for the failure of presurgical fMRI even at 1.5 T (Krings et al., 2001). This study addresses the question of whether activation may be isolated from motion and other artifacts in ultra-high field fMRI using independent component analysis (ICA), and assesses the specificity of activation maps derived with ICA compared with those generated with the general linear model (GLM) approach.

Head motion between image volumes generates signal changes at contrast boundaries such as the ventricles and edge of the brain, while displacement in the slice select direction during one TR leads to spin history effects (Friston et al., 1996). Motion also introduces dynamic non-linear distortions in regions of high susceptibility gradients (Hutton et al., 2002; Robinson and Jovicich, 2011; Visser et al., 2012) and increases Nyquist ghosting and parallel imaging reconstruction artifacts (Poser et al., 2013). Head motion artifacts are particularly severe in patient studies (Bullmore et al., 1999; Seto et al., 2001) and at very high field, as parallel imaging reconstruction artifacts, eddy currents, and B0 changes due to motion increase (Beisteiner et al., 2011).

Head motion can be reduced to some extent using molded cushions (Kearfott et al., 1984) or restraining masks or helmets (Greitz et al., 1980; Fox et al., 1985; Edward et al., 2000). Some residual motion will be present, however, particularly if jaw movement is inherent to the task. While motion can be corrected for prospectively by tracking the head position (Zaitsev et al., 2006; Ooi et al., 2009; Qin et al., 2009), this cannot eliminate effects relating to motion during acquisition of a volume or changes to the shim brought about by a modified head, jaw, or tongue position. Motion-correction algorithms can improve the quality of fMRI results (Oakes et al., 2005) but cannot correct for changing distortions or spin history effects, and can also lead to false positive fMRI results (Wu et al., 1997; Freire and Mangin, 2001). Motion parameters can be included in a GLM as nuisance variables. This reduces motion contamination, particularly in event-related designs (Birn et al., 1999), but substantially reduces BS when even moderate correlation exists between motion and task (Johnstone et al., 2006). In short, while a range of strategies exist to minimize and correct for motion, some level of motion artifacts will remain, particularly in ultra-high field fMRI with tasks which necessitate some motion, such as overt speech (Foki et al., 2008) and motor tasks, particularly of the jaw or feet. If motion is uncorrelated with the stimulus these effects lead to increased residuals after fitting with a GLM, which reduces BS (Friston et al., 1996). If they are time-locked to the stimulus they can lead to false positive results in a GLM (Hajnal et al., 1994).

Spatial ICA is a promising alternative analysis approach to isolating activation in data containing motion effects since it identifies signal sources on the basis of spatial independence rather than the temporal similarity between stimulus and response. As well as proving effective in identifying activation in conventional fMRI experiments (McKeown et al., 1998), ICA can detect BOLD signal changes resulting from epileptic events (LeVan and Gotman, 2009) and multiple neuronal networks to be separated in such challenging contexts as natural stimulation (Malinen et al., 2007) and the resting state (Beckmann et al., 2005).

Independent component analysis has proved capable of separating activation from computer-simulated motion (McKeown et al., 1998). In the context of real motion, however, ICA has, to date, been used as a filtering tool (Kochiyama et al., 2005; Tohka et al., 2008; Kundu et al., 2012) or to motion-correction data (Liao et al., 2006). In this study, we test ICA as the primary means to identify activation in data containing real motion effects. Our study hypotheses were:

1. that ICA would allow a near complete separation of stimulus-correlated motion and activation, even where there are deviations from task timing or modified HRF in the region of pathology and
2. that it would be possible to identify one or more components reflecting task-relevant activation automatically on the basis of temporal and/or spatial characteristics, or “features.”

These hypotheses were tested in a clinical study involving chin and hand motion tasks at very high field.

## Materials and Methods

### Patients

All patients participated in the study, which was approved by the Ethics Committee of the Medical University of Vienna, with written informed consent. In the case of minors this was provided by legal guardians. Patients were referred for functional localization of essential motor cortex (primary hand representation – typically localized in the precentral “knob” (Yousry et al., 1997) and primary chin representation – typically the most lateral and inferior part of primary motor cortex) by physicians who were not involved in this study. Most referrals were for surgical planning prior to excision of a tumor. All patients were in a good general state of health at the time of measurement and were able to perform the tasks. Those patients undergoing chin localizations showed normal masticatory function and those undergoing hand localizations could move the relevant hand against resistance. One patient from the Chin group was excluded due to poor performance (difficulty following task timing). Ten patients remained in the Chin study (age range 8–55 years old, mean age 30 ± 16 years old, 5 females); see Table 1 for demographic and clinical details. The Hand study consisted of 12 patients (age range 11–61 years old, mean age 31 ± 17 years old, 5 females); see Table 1.

**Table 1:** **Patient demographics**.

Patient ID		Head coil (# elements)	Age	Gender	Number of runs completed		Pathology
Chin	Hand				Chin	Hand	
C1	H1	24	55	F	12	7	Left precentral tumor, unknown origin
C2	H2	24	32	F	12	8	Temporal lobe resection left (status post glioblastoma)
C3	H3	24	11	M	10	5	Fronto-central focal cortical dysplasia right
C4	H4	24	21	M	12	8	Right central tumor, unknown origin
C5	H5	8	36	M	12	8	Right frontal tumor, unknown origin
C6	H6	24	28	M	11	8	Oligodendroglioma II., frontal lobe right
C7	H7	32	54	F	10	8	Left parietal tumor, unknown origin
C8	H8	32	14	M	10	8	Extra-temporal epilepsy
C9	H9	32	21	F	12	8	Temporal lobe epilepsy right, status post partial temporal lobe resection right
	H10	24	61	F		4	Suspected precentral glioma right
	H11	24	14	M		10	Cryptogenic epilepsy of the right parietal lobe
	H12	24	21	M		7	Fibrillary astrocytoma (grade 2), temporal lobe epilepsy right
C10		32	8	F	8		Focal cortical dysplasia frontal and occipital

*Patients who performed the chin task have patient IDs beginning with “C” and those who performed the hand task “H.” These IDs are used in other images and descriptions in the text*.

### Tasks

The functional chin paradigm was repetitive opening and closing of the mouth with a target of one open and close cycle per second. The movement was self-paced and symmetrically performed in a blocked design. The hand task was a repetitive opening and closing of the affected hand with the eyes open. For both tasks, each run consisted of four rest and three movement phases of 20 s (eight volumes). Patients were asked to perform 20 runs in total if they were able. If a number of tasks were performed in the same scan session (e.g., chin, hand, foot localizations), the task for each run was communicated prior to the beginning of the run. Commands to begin and stop movement were communicated via headphones during image acquisition.

### fMRI acquisition

Images were acquired with a 7 T Siemens MAGNETOM scanner (Siemens, Erlangen, Germany). Three different head RF coils were used, as hardware upgrades were undertaken during the study. These were an 8-channel coil (Rapid Biomedical, Würzburg, Germany), a 24-channel coil (Nova Medical, Wilmington, MA, USA) and a 32-channel coil (Nova Medical). Table 1 lists which coil was used for each patient measurement. To minimize head movement, plaster helmets were individually constructed for each patient (Edward et al., 2000). Functional MRI data were acquired with a 2D single-shot gradient echo (GE) EPI sequence, with 34 slices acquired parallel to the AC-PC plane, with a matrix size of 128 × 128, FOV = 230 mm × 230 mm (nominal 1.8 mm × 1.8 mm in-plane resolution), 3 mm thick slices with 0.3 mm gap. This EPI protocol has been used in a number of prior studies with neurological patients at 3 T (e.g., Foki et al., 2007; Beisteiner et al., 2010), and has been validated in clinical application at 7 T (Beisteiner et al., 2011). The resolution is in the higher resolution regime in which physiological noise is minimized and the highest BS gains are expected with field strength (Triantafyllou et al., 2005). Three dummy excitations were performed before acquisition of 56 volumes per run. TE/TR were 22/2500 ms, and partial Fourier encoding was used, with omission of the first 25% of phase-encoding steps, receiver bandwidth was 1445 Hz/pixel, and parallel imaging with GRAPPA (Griswold et al., 2002) was used with a factor of 2.

High-resolution T1-weighted MR images were acquired using a 3D MPRAGE sequence with a matrix size of 320 × 320 × 224, with 0.7 mm isotropic resolution, flip angle of 9°, and GRAPPA acceleration factor 2; acquisition time 7 min 57 s.

### fMRI preprocessing

**Figure 1:**
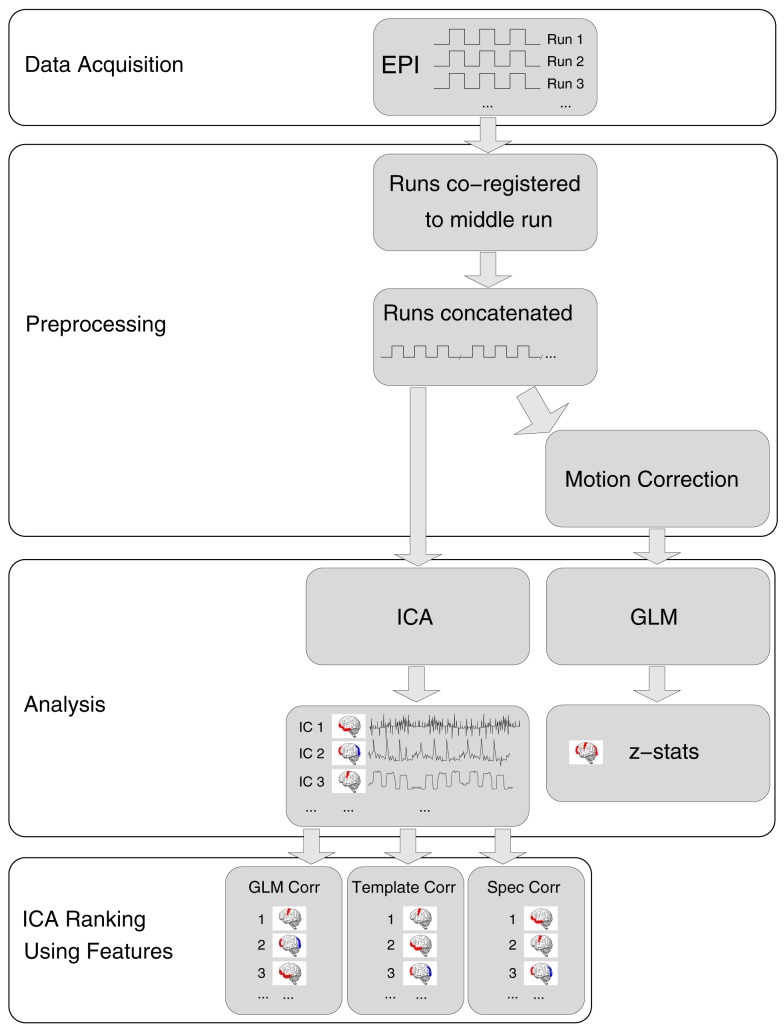
**A schematic representation of data processing steps for each patient in the main analysis**.

Acquisition, preprocessing, and analysis steps are schematically illustrated in Figure 1.

Image preprocessing was carried out in general accordance with the approach used by the Clinical fMRI Study Group at the Department of Neurology of the Medical University of Vienna for presurgical mapping (e.g., Foki et al., 2007; Beisteiner et al., 2010, 2011). For the single-subject analysis, the following preprocessing steps were carried out, with FSL (Smith et al., 2004), in the native space of the high-resolution EPI of each patient. Each run was registered to the first volume of the middle run using FLIRT (Jenkinson et al., 2002), with 12 degrees of freedom, after which runs were concatenated. In the GLM, temporal concatenation equates to a fixed effects analysis in which run is treated as a fixed effect, a valid approach if there is signal stability between runs and no inferences are to be drawn about a group. No slice timing, normalization, or spatial smoothing was performed. These data were analyzed with MELODIC ICA (Beckmann and Smith, 2004). For GLM analysis, the concatenated time-series was additionally motion-corrected using MCFLIRT (Jenkinson et al., 2002). Non-brain tissue was also removed using BET (Smith, 2002), the grand-mean intensity of the entire 4D dataset was normalized using a single multiplicative factor and high-pass temporal filtering was applied (Gaussian-weighted least-squares straight line fitting, with sigma = 20.0 s).

### GLM analysis

Analysis was carried out using FSL’s FEAT (Smith et al., 2004). The six parameter rigid-body transformations determined in motion correction were included in the analysis model as confounds. Time-series statistical analysis was carried out using FILM with local autocorrelation correction (Woolrich et al., 2001). Resulting statistical *z*-images were first thresholded at *Z* > 2.3 to determine continuous clusters. Each resulting cluster was then compared against a (corrected) cluster significance threshold of *P* < 0.05 using Gaussian random field theory (Worsley, 2001).

The possibility the GLM may be able to provide an improved separation of activation and motion-related artifacts at higher statistical thresholds was investigated by closely examining results over a range of thresholds. In supplementary analyses, the possibility of reducing motion-related artifacts in GLM results via cluster size was evaluated by using larger cluster extent thresholds. The possibility that using no cluster thresholding might reveal activation in the basal ganglia and thalamus in GLM results was assessed by applying no cluster extent threshold.

To explore additional possibilities for reducing motion artifacts, GLM analysis was also repeated (i) with the inclusion of the temporal derivatives of motion parameters (in addition to the motion parameters themselves), (ii) on data which were smoothed with a Gaussian kernel with FWHM of 5 mm, and (iii) in a subject-level analysis, rather than a temporal concatenation analysis.

### Independent component analysis

Probabilistic ICA was carried out with FSL’s MELODIC (Beckmann and Smith, 2004). No temporal filtering was performed on the assumption that the signal was stable and that ICA would prove capable of isolating minor drifts, if present, in separate components. Non-brain voxels were masked before voxel-wise de-meaning of the data and a normalization of voxel-wise variance. Pre-processed data were whitened and projected into an *n*-dimensional subspace using probabilistic principal component analysis. The number of components into which the data was decomposed (the model order) was estimated for each patient using the Laplace approximation to the Bayesian evidence of the model order (Beckmann and Smith, 2004).

### Automated identification of salient ICs

Several hundred components may be generated in the analysis of data from each patient. From these, the single component or small number of components which reflect task activation must be identified. In MELODIC, components are ordered by the percentage of the total signal variance in the data for which they account. In the presence of motion and other artifacts, task-related activation often appears low in the list, meaning that a large number of components need to be assessed.

One or more ICs related to task activation were identified by a clinical fMRI expert (RB), who assessed all components for all patients. The identification was based on the presence of clear activation in primary and secondary motor areas, with consideration of the effects of the brain pathology (e.g., cluster divisions), supported by time courses which approximately accorded with that expected from the paradigm, and with reference to the clinical report (the local gold standard) (Beisteiner et al., 2000, 2008).

Automatic identification of task-activation components was implemented via ranking of components on the basis of spatial and temporal features. Three features were implemented. The first was the value of the correlation between each IC spatial map and the GLM t-map (“GLMcorr”). The second was the correlation between each IC spatial map and a mask for the precentral gyrus (“TEMPLATEcorr”). The third feature was the correlation between the frequency distribution of ICs and the frequency distribution of the model regressor (“SPECcorr”). For the third feature, correlation between frequency spectra rather than time courses was used to ensure sensitivity to responses which could be delayed due to modified HRF or late task performance (Moritz et al., 2003), and to reduce sensitivity to low frequency behavior such as drift. All features were programed in MATLAB (Mathworks Inc, Natick, MA, USA).

*GLMcorr* was calculated as the correlation between in-brain voxels in the unthresholded IC maps and the unthresholded *Z*-statistic map for the sole contrast of interest in the GLM, using MATLAB’s “corrcoef” function.

The Harvard-Oxford template (Desikan et al., 2006)^1^ was used for the calculation of the *TEMPLATEcorr* feature. This probabilistic atlas assigns unique numerical labels to 48 cortical and 21 subcortical regions. For the *TEMPLATEcorr* feature, the Harvard–Oxford template was converted to a precentral gyrus mask by converting atlas values of 7 (the template value for the precentral gyrus) to 1, and setting all other values to 0. This mask was registered to the space of each patient’s EPI using a transformation derived as follows. First, the MNI T1 brain (i.e., skull-stripped) template, which is in the same space as the Harvard-Oxford template, was coregistered, using FLIRT (Jenkinson et al., 2002), to patients’ MPRAGE structural scans, which had been bias-field corrected with FAST (Zhang et al., 2001), and skull-stripped using BET (Smith, 2002). This defined the first transformation matrix. Secondly, each patient’s skull-stripped, bias-field corrected MPRAGE was coregistered to the middle EPI of the concatenated time-series. This defined the second transformation matrix. The two transformations were combined to define the transformations from the template space to the space of each patient’s EPI. The correlation between the precentral gyrus of this template and each IC map was calculated.

For the *SPECcorr* feature, the frequency distribution of each IC, calculated using MELODIC, was correlated with the frequency distribution associated with the predicted responses. This latter was calculated as the Fourier transform of the convolution of a regressor for the ON and OFF task periods convolved with a HRF. The HRF was generated with the statistical parametric mapping (SPM) software (Friston et al., 1995) using the function spm_hrf.m, in the SPM8 version^2^, using default values for the parameters (*p*) of the response. The convolution and Fourier Transform were carried out in MATLAB.

### Time-course analysis

To assess the signal behavior in activated areas virtually free from bias of analysis approach (ICA or GLM), the mean time courses (over runs) of voxels in the left and right primary motor areas (PMA) were calculated for each patient, and averaged over runs. VOIs were coboids sized 9 × 9 × 9 voxels centered on the peak voxel in the ICA results in the left and right motor cortex. The ICA results were chosen because they were cleaner, but selection of the GLM peak voxels would not significantly affect results, as these were close to ICA peak voxels, and VOIs were large.

### Independent assessment of activation

To provide an additional means to assess the validity of GLM and ICA results, activation maps were also generated with the “risk map” approach (Beisteiner et al., 2000, 2008); a correlation analysis over a range of thresholds and with shifted regressors to generate a map of a small number of highly reliably activated voxels. This method has been validated via reference to Direct Electrocortical Stimulation (Roessler et al., 2005), and is used locally as a clinical gold standard to generate clinical reports.

## Results

### Extent of head motion

Patients in the Chin group completed between 8 and 12 runs (average 11.0 ± 1.4), and those in the Hand group between 4 and 10 runs (average 7.4 ± 1.6). Rigid-body motion correction yielded three translation vectors (*x*, *y*, *z*) and three rotation vectors (roll, pitch, yaw). These were reduced to two representative metric vectors, one for translation – the root-mean-square (RMS) translation and one for rotation – the sum of the magnitudes of the individual angles (i.e., disregarding sign). Over all patients and all runs in the Chin group, the mean RMS displacement was 0.43 ± 0.45 mm, and the mean rotation 0.0078 ± 0.0083 rad. Corresponding values for the Hand group were a mean RMS displacement of 0.107 ± 0.058 mm and a mean rotation of 0.0035 ± 0.0030 rad.

### Chin task

#### General linear model

**Figure 2:**
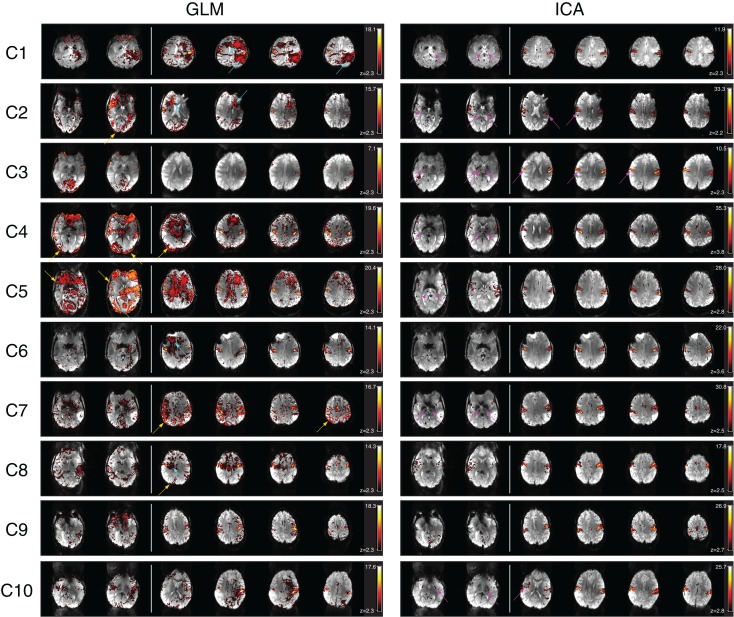
**A comparison of GLM and ICA analyses of 7 T fMRI data with a chin task**. GLM results are contaminated by motion artifacts (yellow and cyan arrows). ICA components show no motion contamination and bilateral activation throughout primary motor areas. Activated areas not present in corresponding GLM results, or not distinguishable from artifacts, are indicated by magenta arrows. White vertical lines separate sample slices covering the basal ganglia from those showing primary motor regions. All brain images are displayed in radiological convention.

There were no significant signal discontinuities between runs. Motion artifacts were identified as suprathreshold voxels either on the edge of the brain or at high contrast boundaries or in areas affected by Nyquist ghosts (Hajnal et al., 1994; Robinson and Moser, 2004; Beckmann, 2012). This attribution was supported by an assessment of the independent components whose time courses correlated best with motion parameters (not shown). Motion artifacts were present in all GLM results at a cluster-corrected threshold of *P* < 0.05 (Figure 2, left). Partial volume motion artifacts manifested as suprathreshold voxels either on the edge of the brain or at high contrast boundaries. These were apparent in the GLM results of patients C2, C4, C5, C7, and C8 (Figure 2, left, at yellow arrows). Broad areas of false positive results, tentatively ascribed to reconstruction artifacts, were present in GLM results of patients C1, C2, C4, C5, C6, and C10 (Figure 2, left, at cyan arrows). Typically these artifacts were reduced at higher thresholds but did not disappear. Increasing the cluster extent threshold did not help to reduce motion artifacts, as they were large and distributed. Inclusion of the temporal derivatives of motion parameters in the analysis (in addition to the motion parameters themselves) led to a moderate reduction in the artifact level in two patients (C7 and C9, not shown) but not in other patients. Smoothing data prior to GLM analysis increased significance values in both artifacts and activation clusters, leaving the overall pattern of suprathreshold voxels smoother, but broadly unchanged. The contamination of GLM results by artifacts was no lower in subject-level analysis than in the temporal concatenation analysis reported here throughout. Basal ganglia activation present in ICA results but not GLM is indicated in Figure 2 by magenta arrows in lower slices.

**Figure 3:**
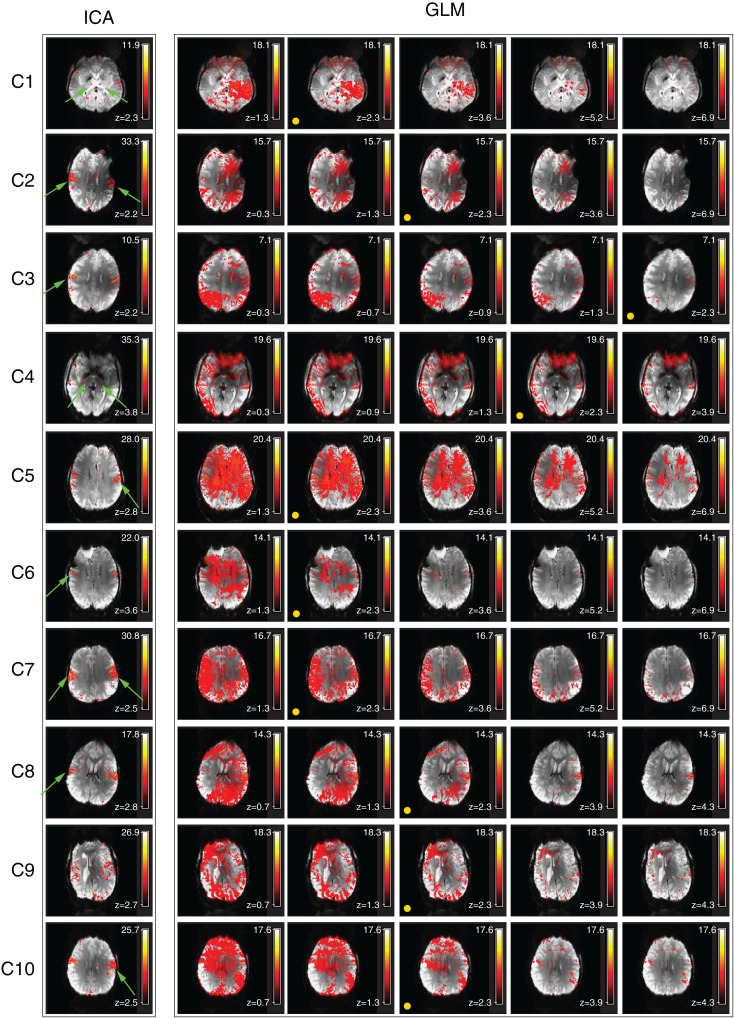
**Examination of the activation visible in GLM results over a range of thresholds (chin task)**. Activation visible in independent components and GLM results is compared in a single slice. The threshold corresponding to a GLM cluster-corrected *P* = 0.05 is indicated by a yellow spot. Activation maps are illustrated at higher and lower thresholds than this to allow the ability to separate activation and motion in GLM results to be assessed. Clusters which are substantially better defined in ICA are indicated by green arrows.

Despite artifacts, it was possible to identify the perirolandic area via detection of central sulcus activation in all patients. Activation was not apparent in some known motor regions, however (Figure 2, magenta arrows). In many patients there was no clearly segregable activation in the basal ganglia and thalamus. The extent to which motion artifacts and low sensitivity to basal ganglia activation may be threshold effects is investigated in Figure 3, and reported in Section “Additional Task-related Components.”

#### Independent component analysis

A task-activation component was identified for all patients. Bilateral precentral gyrus activation was identified in these, with no contamination by motion artifacts (Figure 2, right column). Activation was confirmed to correspond to the clinical report and also to the GLM results (Table 2). Subcortical motor activation, in the basal ganglia, was also present in all patient’s results other than C9 (Figure 2). This was evaluated and judged, on a neuroanatomical and neurophysiological basis, to be plausible task-related activation.

**Table 2:** **A comparison of the ability of the GLM and ICA to detect activation in cortical and subcortical sensorimotor areas in the chin task**.

Patient	Cortical sensorimotor activity (perirolandic cortex)		Subcortical sensorimotor activity (basal ganglia/thalamus)	
	GLM	ICA	GLM	ICA
C1	y	y	(y)	y
C2	y	y	n	y
C3	y	y	(y)	y
C4	y	y	n	(y)
C5	y	y	(y)	y
C6	y	y	y	y
C7	y	y	n	y
C8	y	y	(y)	y
C9	y	y	n	n
C10	y	y	(y)	y

*GLM results were assessed at a number of statistical thresholds. Activation was marked as unequivocally present “y,” arguable “(y),” or not detectable “n.” A clear benefit for detection of subcortical activation was evident with ICA, with better depiction of the basal ganglia in 8 out of 10 patients*.

Separate resting-state networks in motor regions (Biswal et al., 1995) and the basal ganglia (Robinson et al., 2009), which are known from other studies to persist during task execution (Fox et al., 2007; Calhoun et al., 2008) could also be identified in the ICA results of a number of patients (not shown).

#### Comparison of GLM and ICA

Primary motor activation in ICA generally extended into more inferior parts of the motor strip and was more concordant with known motor regions than GLM results, and motion artifacts were dramatically reduced (Figure 2). Basal ganglia activation associated with the motor task was apparent in most patients’ ICA results, but not GLM results. GLM results in the basal ganglia were not substantially changed when no cluster extent size was imposed, regardless of the statistical threshold at which these results were assessed. This demonstrates that the low sensitivity of GLM in subcortical regions was not a cluster extent or a thresholding effect. Thalamic activation was present in all patients’ ICA results other than those of C9, and in the putamen in the results of all patients other than C1 and C9 (Figure 2). Artifacts were also lower in ICA results than in high-threshold GLM images (Figure 3).

For patients C1, C2, C5, C6, C7, C8, and C10, the most inferior and lateral extent of primary motor activation merged with motion artifacts in the GLM analyses, so that their detection was much more difficult, regardless of the statistical threshold. Table 2 lists the extent to which activation could be detected with GLM and ICA in cortical and subcortical regions. GLM and ICA results are compared over a range of GLM thresholds in Figure 3 in slices which indicate increased ICA sensitivity.

### Hand task

#### General linear model

**Figure 4:**
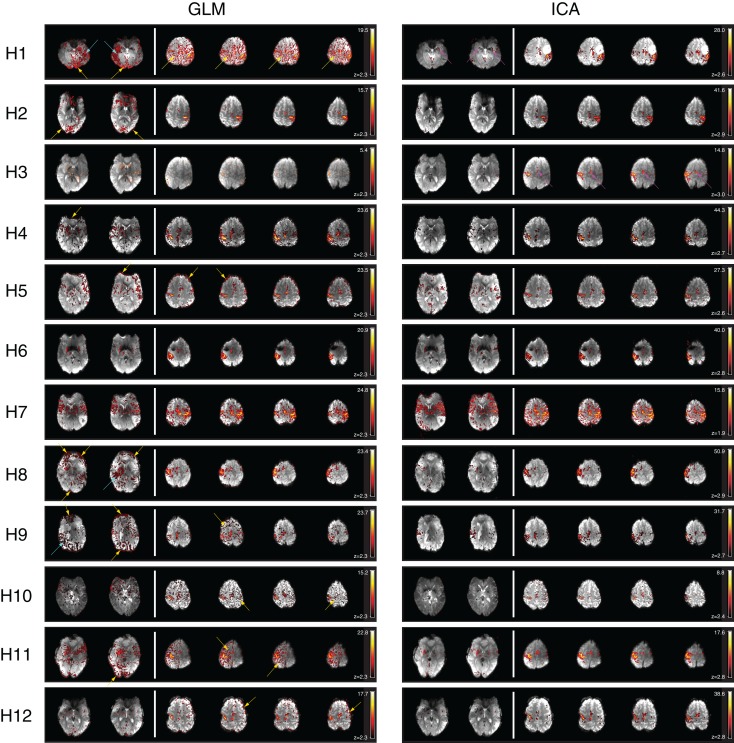
**A comparison of GLM and ICA analyses of 7 T fMRI data with a hand task**. The same thresholds were applied as in Figure 2. Activation in the basal ganglia and thalamus is indicated by arrows in ICA. Activated areas not present in corresponding GLM results, or not distinguishable from artifacts, are indicated by magenta arrows.

General linear model results for patients H1, H2, H4, H5, H8, H9, H10, H11, and H12 were subject to significant contamination by motion artifacts at a cluster-corrected threshold of *P* < 0.05. These artifacts appeared as areas of false positive results on the edge of the brain and/or at boundaries between high contrast areas (Figure 4, at yellow arrows). Wide areas of false positives were also present in H1, H8, and H9 (cyan arrows), originating from reconstruction errors for these GRAPPA-accelerated acquisitions.

Despite contamination by false positive voxels, activation in the contralateral primary motor area, responsible for hand motion, was detected in all the patients. However, activation in the supplementary motor area was difficult to identify due to motion artifacts in H1 and could not be identified in H3 with the GLM at this threshold (magenta arrows). Thalamic activation was present bilaterally in patient H7 and unilaterally in patients H2, H3, H4, H5, H6, H8, H11, and H12. No thalamic activation was evident in patients H1, H9, and H10.

Basal ganglia activation was present in H3, H4, H5, H6, H7, H8, H11, and H12, depicting the putamen either bilaterally (H4, H5, H6, H7, H11, H12) or unilaterally (H3, H8). The posterior part of the left putamen was apparent in H1, but not the right, due to the presence of GRAPPA artifacts. No basal ganglia activation was detected with the GLM in H2, H9, and H10.

#### Independent component analysis

Clear and well-defined activation of the contralateral primary motor area was evident in one or more components in the ICA results for each of the 12 patients. There was little or no motion-related artifact contamination at a canonical Gaussian mixture model threshold of 0.5. Activation in the supplementary motor area was clearly depicted in all the patients.

Activation of subcortical structures, such as the thalamus and the putamen, was evident in the ICA results for all patients except for H9 and H10. A small number of voxels corresponding to activation in the right putamen and in the right thalamus were visible in H3.

Motion artifact level was higher in ICA results in H7 than in other patients, though activation in the primary and supplementary motor regions and in the basal ganglia (putamen and thalamus) was still clearly visible.

#### Comparison of GLM and ICA

There was a high level of consistency in all the patients between PMA identified as being activated using GLM and ICA. Motion-related false positive results were more prominent in GLM results, in which detected activation was in many cases highly contaminated. Motion-related false positives were strongly reduced in both cortical and subcortical regions in ICA results for all patients except for H7, in which the quality of the results was similar in GLM and ICA.

Independent component analysis results for patient H1 show a clear advantage over the GLM results in the depiction of activation in the putamen. The anterior part of the right putamen and the left putamen are easily identified in ICA results, with no surrounding false positives, whereas in the GLM results the anterior part of the right putamen is not visible, even at higher thresholds, and only the posterior part of the left putamen is clearly depicted.

Table 3 details the extent to which activation could be detected with GLM and ICA in cortical and subcortical regions.

**Table 3:** **A comparison of the ability of the GLM and ICA to detect activation in cortical and subcortical sensorimotor areas in the hand task**.

Patient	Cortical sensorimotor activity (perirolandic cortex)		Subcortical sensorimotor activity (basal ganglia/thalamus)	
	GLM	ICA	GLM	ICA
H1	y	y	(y)	y
H2	y	y	(y)	(y)
H3	y	y	(y)	(y)
H4	y	y	y	y
H5	y	y	y	y
H6	y	y	y	y
H7	y	y	y	y
H8	y	y	y	y
H9	(y)	y	n	n
H10	y	y	n	n
H11	y	y	y	y
H12	y	y	y	y

*GLM results were assessed at a number of statistical thresholds. Activation was marked as unequivocally present “y,” arguable “(y),” or not detectable “n.” ICA detected cortical sensorimotor activation in one patient in which it was not clearly visible in GLM (H9), and in the basal ganglia in one patient in which it was not detectable in GLM (H1)*.

### Additional task-related components

**Figure 5:**
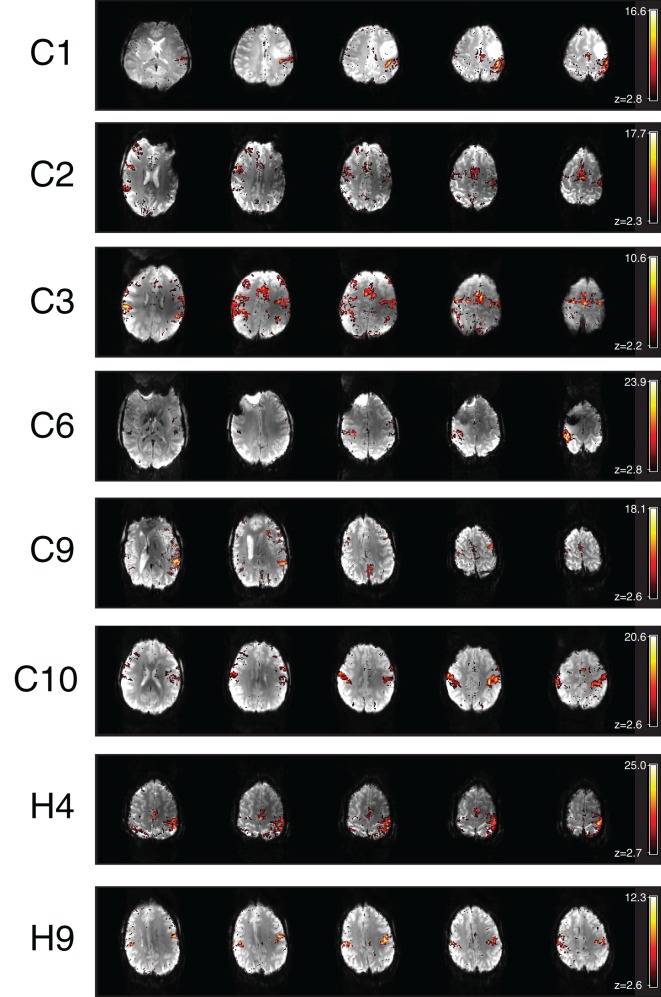
**Additional motor components identified in the ICA results of chin patients C1, C2, C3, C6, C9, C10, H4, and H9**.

**Figure 6:**
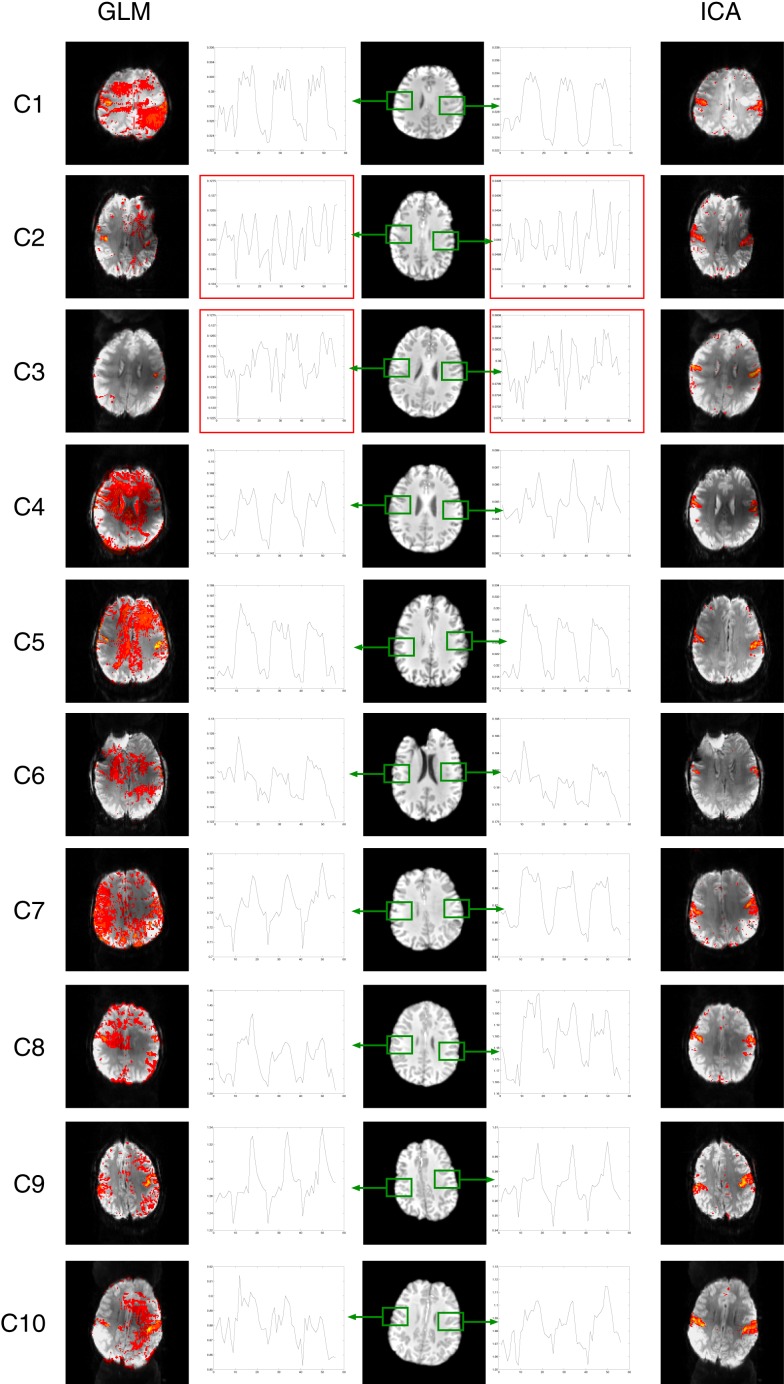
**Mean responses in voxels in primary motor areas in the chin task**. Time courses in PMA in C2 and C3 (outlined in red) are non-model-conform, and correspond with low sensitivity in GLM results (left) but not ICA (right).

For patients C4, C5, C7, and C8, only one component was related to task-related motor activation. For C1, C2, C3, C6, C9, and C10, some task-related activation was present in additional components. In most cases this was secondary motor and basal ganglia activation. These components are illustrated in Figure 5. For C1, a component was identified which showed activation mainly on the side of the pathology, in face-M1. The time course of this second component suggests that it was dominated by activation in a single run. A component for C2 detected secondary motor regions, including the precentral sulcus and SMA, indicating the capacity for ICA to separate subnetworks of motor function. This component was associated with a more rapidly fluctuating time course than the primary component. A component for C3 contained both pre-SMA, SMA, precentral sulcus, and posterior parietal activation, again demonstrating ICA’s ability to separate PMA from secondary areas responsible for motor planning and sensorimotor integration. Neither this nor the primary motor IC showed a time course which correlated well with the stimulus (see Figure 6). An additional component of interest for C6 included participation of the basal ganglia, particularly the thalamus, with activation also in the SMA and right perirolandic area on the pathological side. The time-course of this IC was similar to that of the main component but was dominated by later runs. A secondary component for C9 showed activation in the precentral sulcus and inferior parietal regions, language-related areas, including Wernicke’s area arising from the response to auditory command and possible vocalization. An additional component for C10 showed activation in primary motor area (left hemisphere) and the postcentral sulcus (right hemisphere), reflecting sensorimotor integration (Figure 5).

In H4, the main component shows the predicted activation in the right hemisphere (see Figure 4, which uses radiological convention). An additional task-related motor component was found for patient H4 (see Figure 5), which represents bilateral integrative parietal activity and additional M1 activation in the left hemisphere, in response to motion of the left hand. This could be interpreted as auxiliary M1 activation due to paresis elicited by motion of the contralateral arm. The time course associated with this activation is delayed, so was not detected in the GLM analysis.

An additional bilateral component was also found for patient H9. This was interpreted as representing activation in the face area. The time course of this component is counter to that of the task, indicating that it could be associated with facial movements during the rest phases or with systematic reduction in perfusion in the face area.

### Time courses

With the exception of patients C2 and C3, time-courses in the PMA in the chin task accorded well with the prescribed timing; four rest periods (A) and three task periods (B) of equal duration, presented in an ABABABA design (Figure 6). Time courses in PMA in C2 and C3 are non-model-conform (see graphs outlined red in Figure 6), and GLM results show high levels of noise as well as activation in the PMA. Clean activation is detected in the ICA results, however, indicating that characteristic signal changes take place in the PMA, despite a lack of conformity with the model.

**Figure 7:**
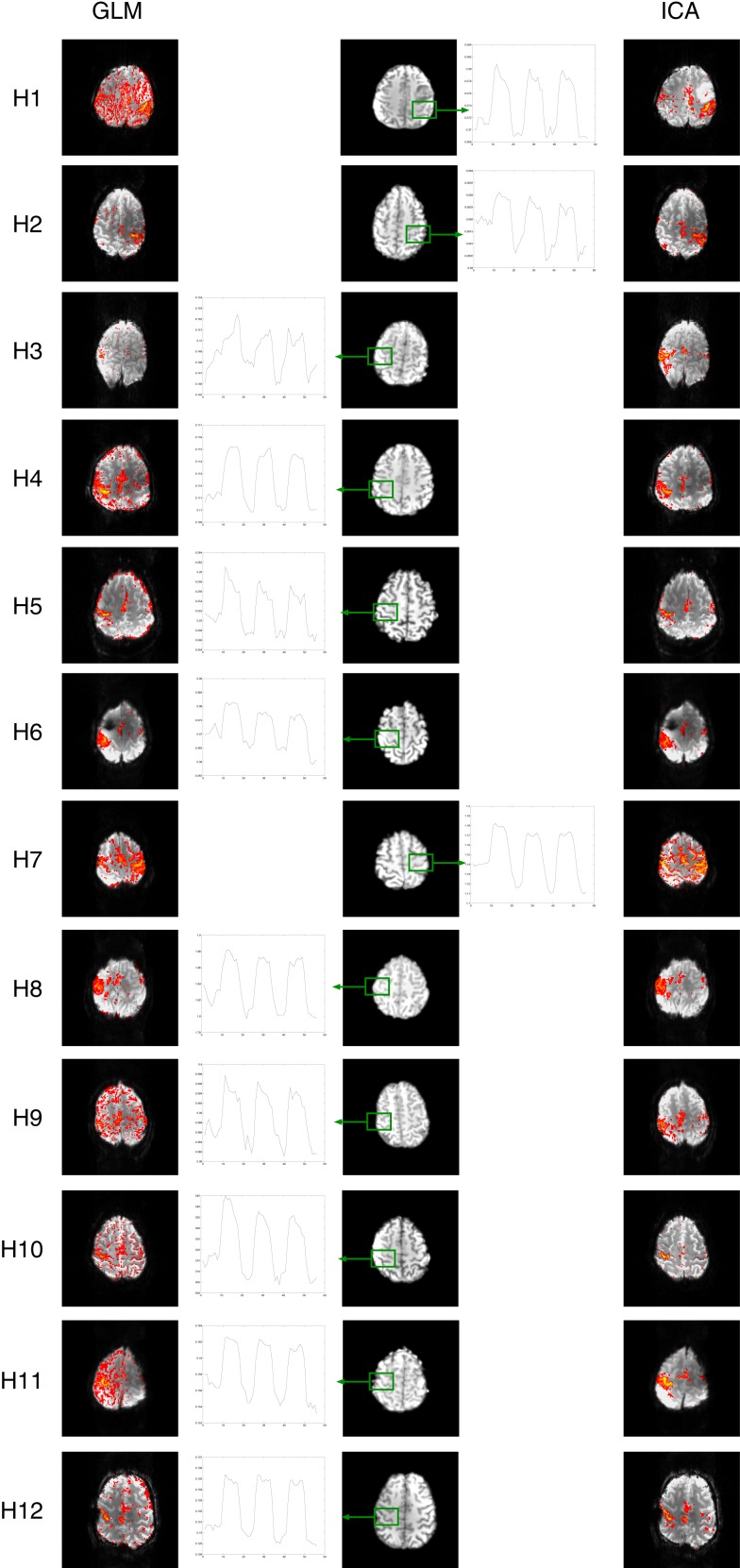
**Mean responses in voxels in primary motor areas in the hand task**. Time courses in PMA in C2 and C3 (outlined in red) are non-model-conform, and correspond with low sensitivity in GLM results (left) but not ICA (right).

Time courses for the hand task were in good agreement with the prescribed timing, which was identical to that in chin task (Figure 7).

### Motion artifacts

**Figure 8:**
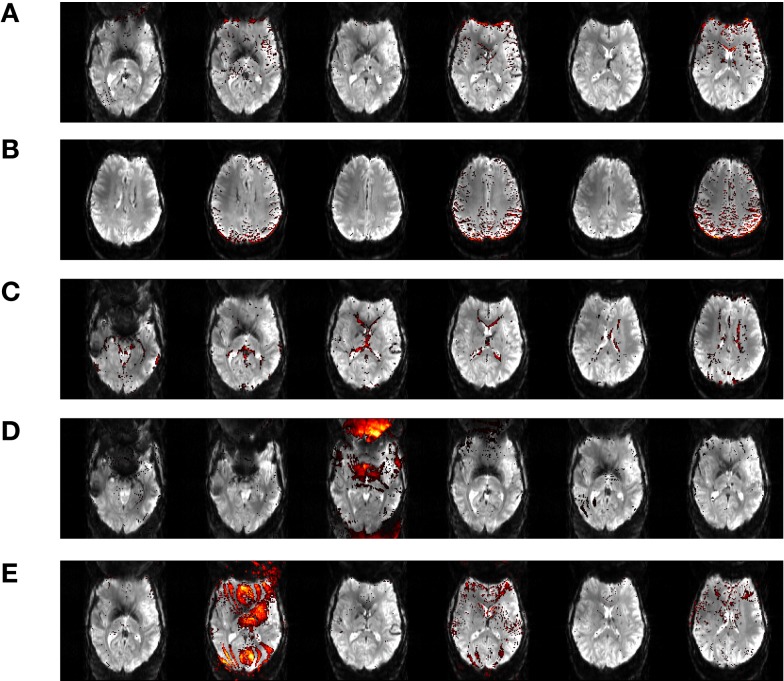
**Motion artifact components isolated in data from Chin patient C5**. These artefacts are attributed to motion in the anterior-posterior direction **(A, B)**, through-plane motion **(C)**, fluctuating Nyquist ghost **(D, E)**.

Independent component analysis allows contributions to the motion artifacts in GLM to be separated into contributing sources and assessed in more detail. We examine these here as an aside from the central aim of this study. Examples of the most prominent artifacts are illustrated in Figure 8, for a single patient, C5, along with tentative attribution of their origin. Artifacts labeled “A” and “B” in Figure 8 arise from motion in the anterior-posterior direction, and manifest at contrast boundaries; the edge of the brain and the borders of gyri. Artifact “C” reflects motion in the through-plane direction, and presents as an outline of the ventricles. One component indicates rapid intensity fluctuations in the Nyquist ghost of a single slice (“D”). Component “E” likewise occurs in a Nyquist ghost region but occurs in every second slice of the volume (which was acquired interleaved), and shows interference with the signal in the main image. These components were identified by their similarity with those reported in Beckmann (2012).

### Automatic identification of salient ICs

**Figure 9:**
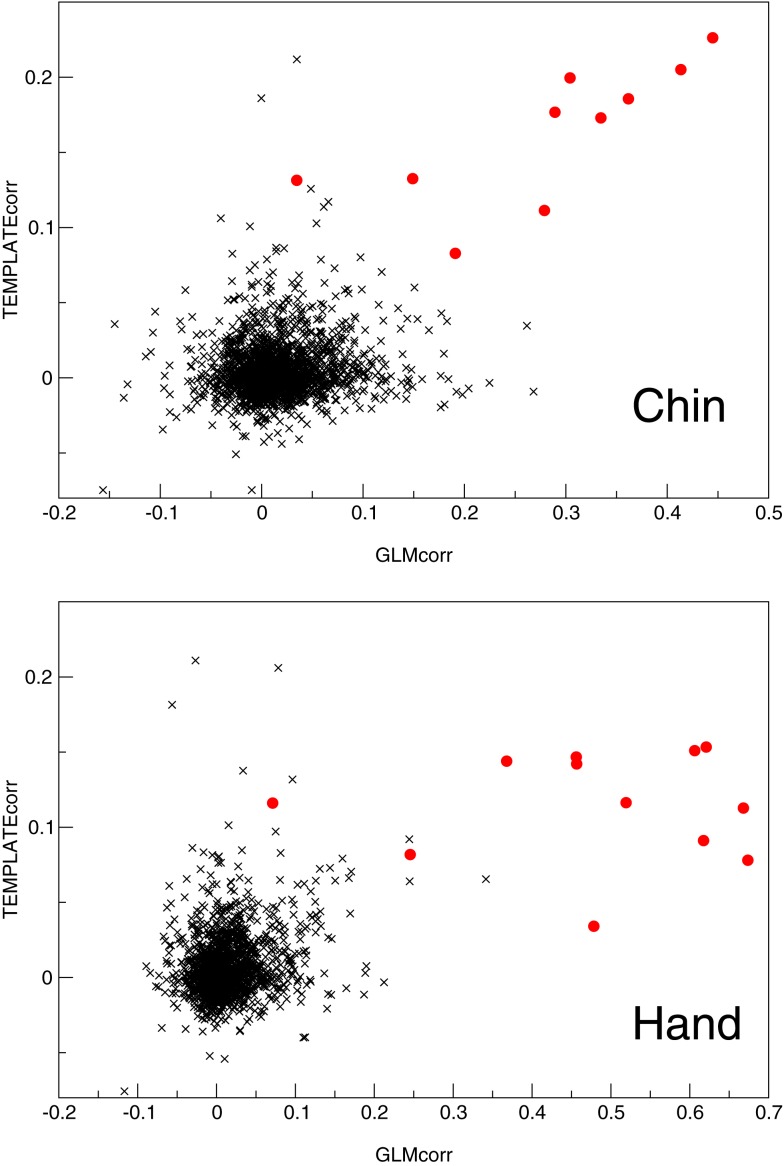
**A plot of the two most successful features used to automatically identify primary motor activation components (red circles) amongst other components (black crosses) in the Chin and Hand groups**.

In MELODIC, independent components are ranked by the percentage of total variance in the data that they explain. Primary task components in the Chin group (which had been identified by an expert) were ranked by variance on average in position 145 ± 48 out of a total of 194 ± 73 components (with quoted errors being one standard deviation). In the single-patient analysis of the hand task, they were ranked in position 92 ± 36 out of 126 ± 43. Primary motor components were ranked more highly using the features tested; GLMcorr, TEMPLATEcorr, and SPECcorr. Of these, both GLMcorr and TEMPLATEcorr were highly effective. Over the Chin and Hand tasks, the primary activation component was ranked in position 1.8 ± 1.0 using TEMPLATEcorr, in position 2.6 ± 6.6 using GLMcorr and 17 ± 47 using SPECcorr. A full list of component rankings by feature is given in Tables 4 and 5 for the Chin and Hand groups, respectively. The potential of the GLMcorr and TEMPLATEcorr features to discriminate from other components is demonstrated in Figure 9.

**Table 4:** **Summary of the total number of components identified in the Chin group (No. ICs) and ranking of the primary motor component in the list by (i) percentage of variance explained (MELODIC default) (ii) GLMcorr: the correlation between IC spatial map and GLM t-map (iii) TEMPLATEcorr: the correlation between IC spatial map and a precentral gyrus template (iv) SPECcorr: the correlation between the frequency spectra of model time courses and frequency spectra of IC**.

Patient ID	No. ICs	Primary motor IC position in ranking by			
		Variance (i)	GLMcorr (ii)	TEMPLATEcorr (iii)	SPECcorr (iv)
C1	234	132	1	2	4
C2	205	191	2	2	18
C3	138	129	32	1	223
C4	200	155	1	1	15
C5	250	159	5	2	16
C6	118	110	1	1	10
C7	209	167	1	1	19
C8	163	162	1	1	5
C9	256	113	1	1	16
C10	165	138	1	1	1

**Median**	**203**	**147**	**1.0**	**1.0**	**15.5**
**Mean**	**194**	**145**	**4.6**	**1.3**	**32.7**
**SD**	**73**	**48**	**9.7**	**0.5**	**67.2**

**Table 5:** **Summary of the total number of components identified in the Hand group (No. ICs) and ranking of the primary motor component in the list by (i) percentage of variance explained (MELODIC default) (ii) correlation between IC spatial map and GLM t-map (iii) correlation between IC spatial map and a precentral gyrus template (iv) correlation between frequency spectrum of model time course and frequency spectrum of IC**.

Patient ID	No. ICs	Primary motor IC position in ranking by			
		Variance (i)	GLMcorr (ii)	TEMPLATEcorr (iii)	SPECcorr (iv)
H1	161	99	1	1	1
H2	138	130	1	3	1
H3	120	80	1	2	44
H4	126	58	1	3	1
H5	169	93	1	6	1
H6	100	70	1	2	2
H7	24	16	1	1	1
H8	158	122	1	3	1
H9	163	108	1	1	1
H10	84	79	1	1	1
H11	159	157	1	1	1
H12	108	102	1	2	1
**Median**	**132**	**96**	**1.0**	**2.0**	**1.0**
**Mean**	**126**	**93**	**1.0**	**2.2**	**4.7**
**SD**	**43**	**37**	**0**	**1.5**	**12.4**

## Discussion

Independent component analysis of 7 T fMRI data acquired from neurological patients performing chin and hand tasks cleanly separated primary motor activation from motion artifacts. Secondary motor areas and the basal ganglia and thalamus could also be distinguished in most patients. A single (default) ICA threshold was appropriate to be able to visualize PMA bilaterally in all patients. GLM analysis of the same data was, in contrast, contaminated by severe motion artifacts arising from partial volume and spin history effects, increased Nyquist ghosting and parallel imaging reconstruction noise. This was despite the use of effective head fixation, motion correction, and the inclusion of motion parameters in the analysis model. Because of these artifacts, GLM results had to be assessed over a range of statistical thresholds in order to be able to identify primary motor activation. The participation of some secondary motor regions and subcortical regions could, in many patients, not be distinguished from artifacts in GLM results. The advantages of ICA were particularly evident in patients whose responses deviated – either because of locally modified hemodynamics or because task execution strayed from the intended task timing – from the model.

Previous studies have used ICA to identify and remove non-activation components (Kochiyama et al., 2005; Tohka et al., 2008). The features implemented included slice-to-slice signal variation, brain boundary signal, and time-course heteroscedasticity, which are very different from the features we applied here, which were targeted at identifying activation rather than artifacts. While artifact removal using ICA was successful in those prior studies at 1.5 and 3 T, it would be substantially more challenging to correctly identify only artifacts in the data acquired in this study, particularly in the chin task. At 7 T, signals resulting from motion – partial volume effects, spin history effects, parallel imaging artifacts, and B0 changes – account for a larger proportion of the total variance than at lower field strength, and motion effects manifest with very different spatial signatures. As such, there is increased likelihood that some motion-related components would not be identified by the algorithms proposed (false negatives), or that activation-related components be erroneously removed (false positives). For that reason, a direct analysis with ICA, with ranking/classification of components, would seem to be a more promising option than filtering with ICA prior to GLM analysis.

The model orders estimated in this study (mean ± SD over patients: 194 ± 73) were much higher than those reported by Tohka et al. (2008). This is likely to be because our concatenated runs were longer and the data itself more complex, due to the artifacts induced by the task combined with higher resolution, the use of GRAPPA, and very high field. In a study into model order, Abou-Elseoud et al. (2010) found that 70 ± 10 components were appropriate for PICA of the 1.5-T data they considered, but that “Different model orders may be found more optimal when higher field strengths and higher resolutions are used.” Our findings support those authors’ conclusions.

Resting-state networks in motor regions (Biswal et al., 1995) and the basal ganglia (Robinson et al., 2009), which are known from other studies to persist during task execution (Fox et al., 2007; Calhoun et al., 2008) (and which are also known as “Temporally Coherent Brain Networks” in this context) could also be identified using ICA in this study. This is of particular relevance for patients who may have difficulty performing motor tasks, as others studies have shown that the sensorimotor area can be localized with resting-state measurements in presurgical populations (Kokkonen et al., 2009).

Recent implementations of ICA (Calhoun et al., 2001; Beckmann et al., 2005) make it a simple analysis to perform. The identification of relevant, activation-related component(s) can, however, be time-consuming, given a large number of independent components. The use of ranking according to one of a number of simple features greatly simplifies this problem. When components were correlated with a precentral gyrus mask, the primary motor component was ranked in position 1 for most patients, and within the first six positions for all others. This reduces the time required for an interpretation of the ICA results by the clinician. A similar approach might also work for other clinical tasks such as presurgical language mapping with neuroanatomical predefinition of Brocas and Wernicke areas. Limitations concern the possibility of missing components related to neuroplastically shifted brain activations or difficulties in defining neuroanatomical regions of interest in largely distorted brains. In these cases, individual screening of all components would probably still be necessary. In an extension of the ranking we have demonstrated, fully automatic identification of the primary motor component might be achieved with a combination of the “GLMcorr” and “TEMPLATEcorr” features using a trained classifier (Tohka et al., 2008; e.g., Soldati et al., 2009), although this would need to be developed with a larger number of data sets for training and testing. The performance of the *SPECcorr* feature, which has shown to be an effective ranking feature in a previous motor task study at 1.5 T (Moritz et al., 2003) was relatively poor in this context. This is due to the similarity between the frequency spectra of activation and stimulus-correlated motion. The poor performance of the *SPECcorr* feature also suggests that the Hybrid ICA approach of McKeown (2000), which combines components with time courses similar to a hypothesized reference function, would be likely to incorporate motion components if applied to these data.

In contrast to Tohka et al. (2008), no motion correction was carried out prior to analysis with ICA in this study. Given effective head restraint, it was expected that ICA would be able to cleanly separate activation from motion-related signal sources without using prior motion correction. This proved to be the case, probably because the dominant motion artifacts in these data were not related to voxel shifts, but rather to changes in B0 and GRAPPA reconstruction errors, which are more pronounced at very high field. At lower field and with less effective head restraint voxel shifts may constitute the dominant source of signal change, and a prior motion correction may be necessary to ensure the effective performance of ICA.

We consider the potential implications of our findings for ultra-high field presurgical planning with motor tasks. In the resection of tumors close to motor regions, the primary aim is to reliably identify the perirolandic area via detection of central sulcus activation. If reliable definition of the course of the central sulcus is possible, the primary motor cortex may be spared in its entirety. In this study, central sulcus activation could be identified in all patients using the GLM approach, despite substantial motion-related artifacts. Depending on the degree of malformations present in the perirolandic area, precentral gyrus regions can be rendered dysfunctional by a tumor, however, and neoplastic reorganization may take place. This leads to function being subsumed by other portions of the precentral gyrus or the contralateral precentral gyrus. In this case it may be necessary to map the primary motor homunculus with a variety of motor tasks. In such cases sensitivity may be required in more inferior regions, where motion artifacts are more pronounced, as observed in this study. ICA results have been shown to be more sensitive and specific in these regions.

The presence of pathology can lead to modification of the hemodynamic response. In presurgical planning it may be necessary to include the temporal derivative of the HRF, use a Finite Impulse Response or Fourier Basis set approach, or estimate the HRF for each patient (Carter et al., 2008; Casanova et al., 2008) and assess the consistency of response over a range of thresholds and runs (Beisteiner et al., 2000, 2010). The results achieved here suggest that the same end – robust results in the case of atypical temporal dynamics – may be achieved using ICA with a much reduced clinical analysis and assessment overhead. “Killer applications” of ICA are those in which task timing cannot be monitored, such as studies of the resting state (Beckmann, 2012). While performance can be recorded for many tasks, such as the simple motor tasks described here, there may be an absence of compatible monitoring devices for ultra-high field systems, and some stages of processing may be hard to monitor for other tasks relevant to presurgical planning, such as the “home town walking” task used to map memory (Beisteiner et al., 2008).

For specific clinical questions targeting responses of all parts of a motor network (e.g., movement disorders) and for research purposes, it is desirable to have the sensitivity to be able to detect the participation of motor regions which may show smaller BOLD signal changes, such as subcortical sensorimotor areas predominantly involved in extrapyramidal motor disease. Our results indicate the most prominent benefit of ICA for such tasks.

Although not directly assessed in this study, the artifacts observed here are expected to be similar to those encountered with overt speech paradigms used in presurgical localization of language (Gartus et al., 2009). Language tasks lead to smaller BOLD signal changes which are localized more inferiorly, where artifacts are more pronounced. Another promising area of application is basic neuroscience studies involving painful or emotionally evocative stimuli, which may likewise elicit substantial motion (Moser et al., 2007). The effectiveness of ICA in isolating the weaker and more variable responses in emotion and language tasks needs to be established in dedicated studies, however.

We have shown that ICA, combined with feature-based ranking of components, constitutes a fast and practical approach to the analysis of 7 T fMRI motor task data containing stimulus-correlated motion. Assessment of the first few ranked components at a single statistical threshold is sufficient to identify motor activation without contamination by motion artifacts, offering additional information and clarity compared to a GLM analysis. ICA allows advantage to be taken of the increased SNR and BS promised by ultra-high field for clinical studies (Beisteiner et al., 2011) even for challenging tasks involving head motion. This paves the way for increased reliability of results and the use of higher resolution in such applications as presurgical mapping at 7 T.

## Conclusion

Independent component analysis was found to be capable of cleanly separating activation from motion artifacts in ultra-high field fMRI data which contained stimulus-correlated motion. Some activated regions were evident in ICA results but not GLM results, indicating not only higher specificity to activation but also higher sensitivity in the analysis of motion-contaminated data. The features presented here allowed task-relevant activation components to be easily identified from the large number of contributing signals, making ICA a feasible approach to the routine analysis of presurgical planning fMRI data with motor tasks in the lab and clinic. The fact the correlation between GLM results and ICA spatial maps allowed the primary motor components to be identified in most patients adds weight to the argument that both methods should be applied to the analysis of such patient data.

## Conflict of Interest Statement

The authors declare that the research was conducted in the absence of any commercial or financial relationships that could be construed as a potential conflict of interest.

## Footnotes

^1^http://www.cma.mgh.harvard.edu/fsl_atlas.html

^2^http://www.fil.ion.ucl.ac.uk/spm/software/spm8/
